# A Preliminary Survey of Autism Knowledge and Attitude among Health Care Workers and Pediatricians in Tehran, Iran

**Published:** 2019

**Authors:** Mohammad EFFATPANAH, Ghazal SHARIATPANAHI, Amirsina SHARIFI, Rozita RAMAGHI, Reza TAVAKOLIZADEH

**Affiliations:** 1 Ziaeian Hospital, Tehran University of Medical Sciences, Tehran, Iran

**Keywords:** Autism spectrum disorders, Attitude, Knowledge, Health care worker, Pediatrician

## Abstract

**Objective:**

Autism spectrum disease (ASD) is not a common diagnosis for children presenting with neurodevelopmental delay before 36 months in Iran. Although recent years have witnessed improvements in diagnosis of pediatrics psychological disorders the role of referral system starting with health care workers (HCW) is not clear. Therefore, we aimed to investigate the common concepts about ASD among pediatricians and HCW.

**Materials & Methods:**

Pediatricians were randomly selected from four teaching hospital of Tehran University of Medical Sciences, Tehran, Iran in 2012-13. HCW were randomly selected from 3 urban health care centers in Tehran, Iran. DSM-IV TR criteria for ASD was used to assess knowledge. Participants were asked to rate sixteen statements on beliefs about autism to assess attitude.

**Results:**

Overall, 122 pediatricians and 90 HCWs with mean ± SD age of 36±4.7 yr and 76.4% being female recruited. Pediatricians had significantly higher encounter with autistic patients (18% vs. 10%, *P-*value=0.06) and parents of autistic child (17% vs. 12%, *P*-value=0.07). But generally, 209 participants (98.6%) declared that they were familiar with autism. There was no statistically significant difference between study groups in rating DSM-IV TR criteria for ASD as “necessary for diagnosis”. Age, gender and working experience, did not differ between pediatricians or HCW answers (all *P*-values >0.05). Among HCW participants, higher educational level was associated with higher disagreement about “autistic children is schizophrenic” (*P*=0.01). Moreover, HCW with higher working experience had higher agreement rate with “autistic children needs special education” statement (*P*= 0.04).

**Conclusion:**

There are still misconceptions about ASD regarding developmental, cognitive and emotional features in both HCW and pediatricians needed to be educated through national program.

## Introduction

Autism spectrum disorders (ASD) are a group of neurodevelopmental disorders characterized by impaired social interaction, disabled verbal and non-verbal communication and repetitive behavior (1). These symptoms begin in infancy or early childhood and last for a lifelong (2). Despite great efforts in improving knowledge and researching on this topic in developing countries, there is still a wide gap in many aspects, especially in knowledge and attitudes of different healthcare providers about ASD (3, 4). Previous studies are suggestive for different level of knowledge about etiology, treatment and prognosis of ASD among general practitioners, pediatricians and neurologists, and child psychiatrists, speech therapists and psychologists (5-8). However, there are two points to be mentioned. First, these studies are mainly from developed countries and in developing countries, due to lack of research infrastructures, there is no properly designed epidemiological study even about prevalence of ASD (3). Second, ASDs are more prevalent in developed countries, raises a question about knowledge and attitude of first-line healthcare workers (HCW) about ASD in developing countries. 

Although Iran is a pioneer in adult psychiatry among Middle Eastern countries, pediatric psychiatric services are not as developed. There is no structured referral system to psychiatric centers for children and the number of specialists in child psychiatry is scarce. In this regard, patients screening and diagnosis relies deeply on HCW and pediatricians, respectively. On the other hand, unfamiliarity with common signs and symptoms of ASD and having misconceptions about etiology and course of disease along with being unaware about DSM-IV TR diagnostic criteria of ASD delays early diagnosis and intervention. Therefore, we aimed to investigate common concepts about ASD among pediatricians and HCW.

## Materials & Methods

This cross-sectional study was conducted on pediatricians and HCW on the autism knowledge and attitude from Jan 2012 to Jan 2013. Study participants were all selected from urban health care center and teaching hospitals related to Tehran University of Medical Sciences, Tehran, Iran. Tehran is the capital city of Iran with estimated population of 12 million people and estimated number of 20 autistic children in every 10,000. Pediatricians were randomly selected from 4 hospitals including Ziaeian Hospital, Bahrami Hospital, Children’s Medical Center and Vali-e-Asr Hospital using cluster sampling method. HCW is called to every person working in an urban health center trained to perform primary health programs. This person might have M.S or B.S in public health but he/she is not a general practitioner or pediatrician. HCW were randomly selected from 3 urban health care center using cluster sampling method. 

The institutional review board and Ethics Committee of Tehran University of Medical Sciences approved the study design. 

The survey form consisted of 3 sections: demographics and experience with autism, diagnostic criteria of autism according to DSM IV-TR (9) and sixteen sentences regarding social, emotional, cognitive, treatment and prognostic attitudes toward autism. Participants were asked to rate diagnostic characteristics of autism as “Necessary”, “Helpful but Not Necessary” or “Not Helpful”. The respondents were informed to rate each statement about their beliefs either as “Agree”, not Sure”, or “Disagree”. 

In order to define these sixteen statements on attitudes toward autism, a version of autism survey (10) was used and then it was modified in focus group discussion including 10 experienced specialists in child neurology and child psychiatry. The internal consistency of this questionnaire was assessed using Cronbach's alpha test and it was 0.8 which provided good reliability. 

Categorical variables are expressed as percentages and frequencies and continuous variables are shown as mean ± standard deviation. Categorical and continuous variables were compared using the chi-squared test and the independent student t-test, respectively. All analyses were performed by the two-sided method using SPSS version 22 (Inc., Chicago, IL), and the *P*-value of <0.05 was set as statistically signiﬁcant.

## Results

Three hundred questionnaires were distributed of which 250 were returned showing a response rate of 83.3%. However, 38 questionnaires were incomplete and excluded. The final number of participants were 212. Mean ± participant`s age was 36±4.7 yr and 63.2% were 30-40 yr. Most of the participants were female (76.4%) and 60.8% of participants were married. Overall, 122 pediatricians and 90 HCW were recruited. The working experience was categorized to 0-10, 10-20 and >20 yr and 77.8%, 16.5% and 5.7% of participant were in these categories, respectively. Pediatricians had significantly higher encounter with autistic patients (18% vs. 10%, *P*=0.06) and parents of autistic child (17% vs. 12%, *P*=0.07). However, generally 209 participants (98.6%) declared that they are familiar with autism. 

Table 1 demonstrates pediatricians and HCW answers to listed ten characteristics of autism according to DSM IV-TR diagnostic criteria. 

We assessed the answer of pediatricians and HCW to diagnostic criteria of autism in different age subgroups (<30, 30-40 and >40 yr), gender (male and female), marital status (married, single and divorced) and working experience (10>, 10-20 and >20). None of these factors influenced pediatricians and HCW answers (all *P*>0.05). 

Table 2 summarizes pediatricians and HCW beliefs about autism. Only the absolute counts and related percentage of frequency of being “Agree” to the statements are presented in order to minimize the table.

As we assessed pediatricians and HCW beliefs in different subgroups of age (<30, 30-40 and >40 yr), gender (male and female), marital status (married, single and divorced) and working experience (10>, 10-20 and >20) only following relations were statistically significant: “Autism separates children from parents” and marital status (*P*=0.02, married participants represented higher agreement rate), “autistic children need special education” and gender (*P*=0.04, male participant had higher disagreement rate), “autistic children needs special education” and working experience (*P*=0.04, participants with higher working experience had higher agreement rate), “autistic children are mentally retarded” and marital status (*P*=0.05, married participants represented higher disagreement rate), “autism is neurodevelopmental disorder” and gender (*P*=0.03, male participant had higher disagreement rate).

Among HCW participants, higher educational level was associated with higher disagreement about “autistic children is schizophrenic” (*P*=0.01).

**Table 1:** Comparative percent of pediatricians and HCW answers to diagnostic criteria of autism

Diagnostic characteristics	pediatricians			HCW			*P *value
	Necessary (%)	Helpful but Not Necessary (%)	Not Helpful (%)	Necessary (%)	Helpful but Not Necessary (%)	Not Helpful (%)	
Social interaction difficulties	77(63.1)	41(33.6)	4(3.3)	53(58.9)	32(35.6)	5(5.6)	0.6
Lack of social responsiveness	41(33.6)	62(50.8)	19(15.6)	28(31.1)	44(48.9)	18(20)	0.6
Lack of eye contact	54(44.3)	55(45.1)	13(10.7)	43(47.8)	43(47.8)	4(4.4)	0.2
Language delays	45(36.9)	66(54.1)	11(9)	39(43.3)	47(52.2)	4(4.4)	0.3
Rigid or stereotyped play activities	34(27.9)	68(55.7)	20(16.4)	30(33.3)	49(54.4)	11(12.2)	0.5
Onset of symptoms before 36 months	59(48.4)	54(44.3)	9(7.4)	42(46.7)	43(47.8)	5(5.6)	0.8
Need for sameness, resistance to change in routines	44(36.1)	63(51.6)	23(12.3)	36(40)	46(51.1)	8(8.9)	0.6
Unusual mannerisms such as finger flicking	65(53.3)	51(41.8)	6(4.9)	50(55.6)	38(42.2)	2(2.2)	0.5
Peculiar speech characteristics	35(28.7)	72(59)	15(12.3)	34(37.8)	47(52.2)	9(10)	0.3
Preoccupation with objects	54(44.3)	60(49.2)	8(6.6)	45(50)	42(46.7)	3(3.3)	0.4

HCW: health care workers

**Table 2:** Comparative beliefs of pediatricians and HCW about autism

Belief	Pediatricians (%)	HCW (%)	*P* value
Autism separates children from parents	50(41)	44(48.9)	0.4
Autism happens in families with high socioeconomic status	24(19.7)	25(27.8)	0.3
Autism happens in children of parents with higher educational level	13(10.7)	16(17.8)	0.3
Autistic children are not affectionate	22(18)	10(11.1)	0.1
Autistic children is schizophrenic	13(10.7)	14(15.6)	0.5
It is difficult to differentiate autism from schizophrenia	10(8.2)	9(10)	0.6
Autistic children are neglected from parents	21(17.2)	8(8.9)	0.05
Autistic children needs special education	9(7.4)	15(16.7)	0.1
Autism is a social stigma	13(10.7)	9(10)	0.8
Autism discriminates affected child	14(11.6)	10(11.1)	0.3
There is a general negative view about autism	12(9.8)	11(12.2)	0.6
Parents know symptoms of autism	12(9.9)	7(7.8)	0.8
Autism is preventable	13(10.7)	2(2.2)	0.06
Autistic children are mentally retarded	3(2.5)	2(2.2)	0.4
Autism is neurodevelopmental disorder	82(67.2)	64(71.1)	0.5
Autism is an epileptic disorder	13(10.7)	17(18.9)	0.2

## Discussion

This is the first study investigating and comparing the knowledge and attitude of HCWs and pediatricians about ASD in Iran. HCWs have almost the same attitude and knowledge as pediatricians. ASD prevalence in Iran is not as common as western countries, pediatricians may confront few cases in their residency or their practice. A recent report about the prevalence of ASD has claimed that 86.5% of cases are identiﬁed in three geographical regions of North America, Europe, and Japan and only 10% of the world’s children are from these regions (11, 5). This discordancy emphasizes the big difference in research infrastructures of developed and developing countries. 

Another aspect needed to be considered is that disparity between research facilities of developing and developed countries might have underrated fundamental differences in cultural and social variability, genetic etiology and natural history of autism as well as the potential contribution of environmental exposures in disease prevalence. Moreover, current diagnostic guidelines are all based on researches conducted in developed countries (12, 13). Given these differences, applicability of current guidelines and diagnostic tools should be tested across cultures and social classes of developing countries. 

One of the hallmarks of ASD diagnosis is that symptoms initiate before 36 months of age. However, in both groups of HCWs and pediatricians lower than 50% of participants considered this criterion to be necessary to diagnose the disease. Parents of children with ASD had noticed that there might be “something wrong” with their children in age of fifteen to nineteen months old but as they referred to health professionals, they were reassured to have normal children (14, 15). This is also the problem reported in a study from Pakistan (4). Delay in neurocognitive developmental milestones especially speech is the initial presentation in many known cases of ASD. On the other hand, nonspecific symptoms may be seen in normal patients as well. This lack of knowledge about characteristic presentations and differential diagnosis postpone timely diagnosis.

Both groups have shown different views about ASD compared to previous studies. HCWs and pediatricians were more accurate about the difference between ASD and schizophrenia. Both physicians and non-physicians had misconceptions about natural course of ASD as they believed that an autistic child is more likely to grow up to be an adult with schizophrenia (4). Although there are studies to support this idea (16) other researchers reported less likelihood of autistic features in patients with schizophrenia (17). General practitioners in Karachi, Pakistan have misconceptions about the role of socio-economical class, parental education and cold parenting interaction as etiologic factors for ASD (3). Responders in our study choose higher negative impact for aforementioned factors. 

The pharmacists’ awareness, knowledge, and attitude about childhood autism were investigated in Istanbul, Turkey and reported that most participants believed that ASD holds a stigma in community (6). Parents of autistic children are more vulnerable to avoidance, hostile staring and rude comments from others (18). In our study, both HCWs and pediatricians agreed that ASD causes social stigmatization but the level of agreement was lower than previous studies. 

Our study had few limitations. First, we did not ask about the source of information on ASD in each group. Moreover, we did not ask if education curriculum during study course had helped participants to know autism better. Future studies should be dedicated to assessing the role of attending to medical course about ASD on post-graduation knowledge and attitude of general practitioners and pediatricians. Second, the sampling strategy may not provide a random sample of all HCWs and pediatricians because responders are those who accepted to participate in the study. However, this is an inevitable bias which happens in questionnaire-based studies. 

**In conclusion, **the present study emphasizes on the misconceptions exiting among HCWs and pediatricians about ASD. As HCWs are the first line encounters in urban health care centers and pediatricians are responsible for proper patient selection, management and referral, specially designed curriculum for ASD should be developed to complete effective autism surveillance.
